# The Serum High-Sensitive C Reactive Protein and Homocysteine Levels to Evaluate the Prognosis of Acute Ischemic Stroke

**DOI:** 10.1155/2007/15929

**Published:** 2007-04-10

**Authors:** Tahir Yoldas, Murat Gonen, Ahmet Godekmerdan, Fulya Ilhan, Ednan Bayram

**Affiliations:** ^1^Department of Neurology, Medical Faculty, Firat University, 23119 Elazig, Turkey; ^2^Department of Immunology, Medical Faculty, Firat University, 23119 Elazig, Turkey; ^3^Department of Cardiology, Erzurum Informary Hospital, 25100 Erzurum, Turkey

## Abstract

Ischemic stroke is one of the most common causes of death worldwide and is most often caused by thrombotic processes. We investigated the changes in hsCRP and homocysteine levels, two of these risk factors, during the acute period of ischemic stroke and evaluated the relationship between these levels and the short-term prognosis. HsCRP and homocysteine levels were measured at the 2nd, 5th, and 10th days in forty patients admitted within second of an ischemic stroke. The clinical status of the patients was simultaneously evaluated with the Scandinavian stroke scale. The results were compared with 40 healthy control subjects whose age and sex were matched with the patients. The mean hsCRP levels of the patients were 9.4 ± 7.0 mg/L on the 2nd day, 11.0 ± 7.4 mg/L on the 5th day, and 9.2 ± 7.0 mg/L on the 10th day. The mean hsCRP level of the control subjects was 1.7 ± 2.9 mg/L. The mean hsCRP levels of the patients on the 2nd, 5th, and 10th days were significantly higher than the control subjects (*P* < .001). The patients' mean homocysteine levels were 40.6 ± 9.6 *μ*mol/L on the 2nd day, 21.7 ± 11.1 *μ*mol/L on the 5th day, and 20.7 ± 9.2 *μ*mol/L on the 10th day. The mean homocysteine level of the control subjects was 11.2 ± 1.1 *μ*mol/L. The homocysteine levels of the patients were higher than the control subjects at all times (*P* < .01). In conclusion, patients with stroke have a higher circulating serum hsCRP and homocysteine levels. Short-term unfavorable prognosis seems to be associated with elevated serum hsCRP levels in patients with stroke. Although serum homocysteine was found to be higher, homocysteine seems not related to prognosis.

## 1. INTRODUCTION

Acute ischemic stroke develops as a result of a sudden interruption in the focal cerebral blood flow [1, 2]. The cause of the stroke is an embolic or thrombotic occlusion in 70–80% of patients with severe symptoms [3, 4].

Recent research has shown that an inflammatory reaction is triggered within hours in the brain tissue injured by an ischemic stroke and continues in the days following the appearance of symptoms and that this reaction contributes to neuronal damage [5].

Increased CRP levels are accepted as a sensitive but nonspecific marker of the acute inflammatory response. CRP levels can increase 100 to 500 folds in acute inflammatory conditions [6]. Laboratory and experimental findings have shown that atherosclerosis is a reflection of a chronic inflammatory process in addition to lipid deposition [7, 8]. Inflammatory mechanisms have been known to play a role in all stages of atherosclerosis, from initiation to development [9–12].

It has been reported that it is possible to use the increase in the concentration of acute phase reactants and especially the high sensitivity C-reactive protein (hsCRP) to help predict future cardiovascular morbidity [8, 12–14]. Various prospective studies have found initial CRP levels to be higher in persons who develop stroke, ischemic heart disease, and peripheral artery disease [15–17].

There is increasing evidence that mild hyperhomocysteinemia is an independent risk factor for atherosclerosis and atherothrombosis in the coronary, cerebral, and peripheral vascular structures [17–20].

The aim of this study was to determine the acute course of hsCRP and homocysteine levels following a stroke and their association with the short-term prognosis. An increasing amount of evidence shows these substances to be risk factors for atherosclerosis and therefore ischemic stroke.

## 2. MATERIAL AND METHOD

A total of 40 patients whose age mean is 69 ± 11 years presenting at the Firat University Medical Faculty Neurology Outpatients Department with a clinical picture of acute ischemic stroke within the first 2 days and admitted with a diagnosis of ischemic stroke were included in the study. The control group consisted of 40 healthy volunteers whose age mean is 70 ± 9 years who did not have any defined illnesses and were matched for age and sex with the patient group. The patient and control group subjects were informed on the study, and written consent was obtained.

A diagnosis of acute ischemic stroke was made following a full neurological evaluation including the use of neuroradiologic imaging such as CT and/or MRI in patients who had suffered an acute focal neurologic deficit for more than 24 hours and who did not have a cause other than cerebrovascular disease for this neurological deficit. The clinical status of the patients was evaluated with the Scandinavian stroke scale.

The medical history was obtained from the patients with particular attention to defined risk factors such as DM, smoking, and dyslipidemia. Physical and neurological examinations, CT and carotid-vertebral Doppler ultrasonograpy, and other routine laboratory analyses were carried out on all patients. Patients whose neurological symptoms improved within 24 hours, those receiving a diagnosis of hemorrhagic cerebrovascular disease following clinical and neuroradiologic evaluation and those who had previously suffered an ischemic stroke were not included in the study.

Patients who had heart disease which could lead to cardioembolism such as atrial fibrillation or cardiac valve disease or thyroid or renal dysfunction or kidney failure, patients with local or systemic infection or those who had suffered an infection within the last month, and those with romatoid arthritis, osteoarthritis, or malignancy were not included in the study. The patients were asked about the usage of drugs such as vitamin combinations, methotrexate, tamoxifen, nitrous oxide anesthesia, or anticonvulsant drugs within the last month as these could influence homocysteine concentrations.

Venous blood samples for hsCRP and homocysteine measurements were obtained from the patients on 2nd, 5th, and 10th days. The serum was separated within the hour by centrifugation at 5000 rpm for 10 minutes. The separated sera were kept at −70°C until the laboratory evaluation. The control group provided a single venous blood sample.

HsCRP was studied by the nefelometric method with an hsCRP kit (Dade Behring. USA) on the Behring Nephelometer 100 Analyzer and the results were expressed as mg/L. The limit of detection for CRP is 0.175 mg/L. The homocysteine level was studied with fluorescent detectors using the “high performance liquid chromatography (HPLC)” method with the Clin Rep kit HPLC, Recipechemicals-Instruments GmbH Labortechnik, Munich, Germany and the results were expressed as *μ*mol/L. The determination of homocysteine in plasma is linear within a range of 1 to100 *μ*mol/L and lower detection limit is 0.5 *μ*mol/L.

The results of the groups were expressed as mean ± standard deviation. The data produced during the study were evaluated on the SPSS 12.0 package software program using the Mann-Whitney U test, chi square test, Spearman correlation test, and Kruscal-Wallis test. A *P* value < .05 was considered significant.

## 3. RESULTS

Seven of the 40 patients died during the study period. Fifteen of the remaining 33 patients were able to walk independently at the end of the study period (group A) while 18 patients could not (group B). The 2nd day's SSS scores of died patients (13.7 ± 5.7) were significantly lower than living patients (28.3 ± 14.7, *P* < .05). SSS scores of patients increased gradually and the highest mean scores were determined at the 10th day of illness (36.0 ± 16.0, *P* < .05). The increase in the SSS score among the evaluation days was statistically significant (*P* < .05). The mean SSS scores of patients were shown in Table 1.

The hsCRP levels following the stroke increased from the 2nd day to the 5th day in a significant manner (*P* < .05) and then decreased on the 10th day again in a significant manner (*P* < .05). There was no significant difference between the hsCRP levels in the 2nd day and on the 10th day for hsCRP levels.

When the patient's clinical status according to the 2nd day hsCRP levels was checked, the dying patients had the highest level (14.7 ± 7.9 mg/L) while patients who could walk had the lowest level (7.1 ± 3.9 mg/L) with patients who could not walk having values between these two levels (9.2 ± 8.2 mg/L). The hsCRP level was significantly lower in the patients who could walk compared to the dying patients (*P* < .005). When the 2nd day's hsCRP levels of the dying and surviving patients were compared, the values were significantly higher in the dying patients (14.7 ± 7.9 mg/L) compared to the surviving patients (8.2 ± 6.6 mg/L, *P* < .05). The mean hsCRP concentration was shown in Table 2.

The difference between the three mean homocysteine concentrations of the patient group was not statistically significant (*P* > .05). There was no statistically significant difference between the mean homocysteine concentrations of the dying and surviving patients. It was found that 72.5% of the patient groups have a homocysteine levels higher than 15 *μ*mol/L while it was higher only 25% of the control group and all of the three mean homocysteinemia concentration of the patients with ischemic stroke were significantly higher than in healthy control. There was no significant difference between the female and male patients for homocysteine and hsCRP levels. The mean hsCRP homocysteine concentration was shown in Table 3.

No significant correlation was found between homocysteine and hsCRP levels.

## 4. DISCUSSION

The most important cause of ischemic strokes is atherothrombotic events. Atherosclerosis develops during a long asymptomatic period and the first sign of its existence is an acute event (MI, stroke) [11]. Laboratory and clinical evidence has shown that systemic inflammation plays a role in every stage from the beginning atherosclerosis to subendothelial lipid deposition [8, 11]. HsCRP is an indication of the inflammatory response to atherosclerosis. It is also a response to inflammation that developing during ischemic stroke as a result of tissue injury [21]. Beamer et al. [22] have reported that stroke patients without infection have increased levels of CRP. Experimental stroke studies have shown that secretion of inflammatory mediators as a direct response to cerebral injury starts within two hours of focal ischemia and that occurred anti-inflammatory treatment is also neuroprotective [23–25].

Muir et al. [26] have shown that CRP levels within the first 72 hours following an acute ischemic stroke are an independent indicator for predicting survival. Our prospective study showed that the second day hsCRP levels of patients who died within the first 10 days were significantly higher than the surviving patients. It has been reported that a large infarcts and cortical involvement in patients had a higher CRP values than normal at the time of presentation [27]. The same report also suggests that the prognosis is worse in patients with increased CRP levels. Our finding that patients who could walk independently at the end of the study period have the lowest hsCRP value is also consisted these reports. The cause of the increased hsCRP in patients who died, and the lowest hsCRP levels in the walked group may be a indicator of the degree of underlying inflammation. Under the light of our observation and recent literature, our suggestion is that hsCRP may be a prognostic marker of the prognosis of these patients.

In this study, it was found that 72.5% of the patient group have a homocysteine levels higher than 15 *μ*mol/L while it was higher only 25% of the control group and all of the three mean homocysteinemia concentration of the patients with ischemic stroke were significantly higher than in healthy control. Recent studies have shown that acute hyperhomocysteinemia leads to endothelial dysfunction, contributes to atheroma development [28, 29], and correlates with cerebral arterial stenosis [30].

Meiklejohn et al. [31] reported that the homocysteine levels measured in patients with ischemic stroke and transient ischemic attack in the first 24 hours and after three months did not increase in the acute stage after the atherothrombotic stroke but increased during convalescence. The homocysteine concentrations of the patients were found to be significantly higher than the control group. They have explained this change in the homocysteine levels as due to an increase in the homocysteine level in the prestroke periods which then decrease in the acute phase for unknown reasons. They postulate that this may be a result of changes in the synthesis of plasma proteins in association with the acute phase response [31]. However, it is necessary to check homocysteine levels before and after the stroke to support this hypothesis and it is not possible to predict the development of stroke.

There are several reports suggesting that mean homocysteine concentrations following a stroke are higher in the convalescent period than the acute stage, and mild hyperhomocysteinemia increased the unfavorable prognosis risk by 11.78 times [32–34]. It seems that increased homocysteine levels in these patients may be both a cause and also a result of this process due to acceleration of oxidative stress [32].

In the view of the findings of the present study there was no significant increase in the homocysteine concentrations in the 10 days following a stroke. We conclude that homocysteine levels have a limited value for observation of severity of stroke. Although serum homocysteine was found to be higher, homocysteine seems not related to prognosis. Patients with stroke have a higher circulating serum hsCRP and homocysteine levels. Short term unfavorable prognosis seems to be associated with elevated serum hsCRP levels in patients with stroke and may also be able to predict progression of stroke.

## Figures and Tables

**Table 1:** Mean SSS scores of patients^(a)^.

Mean SSS score	Patients	Group A (independent walking)	Group B (nonwalking)	Group C (died patients)
	*n* = 40	*n* = 15 (37.5%)	*n* = 18 (45%)	*n* = 7 (17.5%)

2nd day	26.4 ± 15.0*	37.3 ± 14.1*	20.8 ± 10.2	13.71 ± 5.7
5th day	31.7 ± 16.0*	45.8 ± 10.7*	24.3 ± 10.7	15.06 ± 3.7

^(a)^Mean ± standard deviation, *P < .05.

**Table 2:** Serum hsCRP levels of study and control groups^(a)^.

hsCRP (mg/L)	Patients *n* = 40	Control *n* = 40	Clinical status		*P* value

2nd day	9.4 ± 7.2	—	Died patients *n* = 7	14.7 ± 7.0*	*P* < .05
5th day	11.0 ± 7.4	—	Independent walking *n* = 15	7.1 ± 3.9	NS
10th day	9.2 ± 7.0	—	Nonwalking *n* = 18	9.2 ± 8.3	NS
Mean	9.8 ± 0.9	1.7 ± 2.9**	—	—	*P* < .001

^(a)^Mean ± standard deviation, NS: no significant.

**Table 3:** Serum homocysteine levels of study and control groups^(a)^.

Homocysteine (*μ*mol/L)	Patients *n* = 40	Control *n* = 40	Clinical status		*P* value

2nd day	20.6 ± 9.6	—	Died patients *n* = 7	20.3 ± 8.2	NS
5th day	21.7 ± 11.1	—	Independent walking *n* = 15	21.2 ± 10.1	NS
10th day	20.7 ± 9.2	—	Nonwalking *n* = 18	20.5 ± 7.2	NS
Mean	21.0 ± 0.6**	11.2 ± 1.1	—	—	*P* < .001

^(a)^Mean ± standard deviation, NS: no significant.
